# Shared decision-making among older adults with multimorbidity in Kerala’s primary care: a qualitative study using the socio-ecological model

**DOI:** 10.3389/fpubh.2025.1665368

**Published:** 2025-09-25

**Authors:** Abhijith A. Kumar, Asha Kamath, Lena Ashok, Veena Ganesh Kamath, Varalakshmi Chandra Sekaran

**Affiliations:** ^1^Department of Applied Statistics and Data Science, Prasanna School of Public Health, Manipal Academy of Higher Education (MAHE), Manipal, India; ^2^Department of Social and Health Innovation, Prasanna School of Public Health, Manipal Academy of Higher Education (MAHE), Manipal, India; ^3^Department of Community Medicine, Kasturba Medical College, Manipal Academy of Higher Education (MAHE), Manipal, India; ^4^Department of Global Public Health Policy and Governance, Prasanna School of Public Health, Manipal Academy of Higher Education (MAHE), Manipal, India

**Keywords:** older adults, multi-morbidity, shared decision-making, person-centered care, treatment burden, health system, non-communicable disease

## Abstract

**Introduction:**

Multimorbidity is an increasing public health challenge among older adults, particularly in Kerala, India. Shared decision-making (SDM) is central to person-centered care for this group, yet its implementation remains inconsistent in low- and middle-income countries (LMICs).

**Methods:**

This qualitative study explored the experiences and perspectives of older adults with multimorbidity regarding SDM in Kerala’s primary care. Sixteen adults (aged 60+) were recruited from four primary health centers using purposive sampling. The Socio-Ecological Model guided the design and thematic analysis, examining SDM influences at individual, interpersonal, organizational, and sociocultural levels.

**Results:**

Key findings revealed that individual barriers, such as limited health literacy and low self-efficacy, led to passive participation. Family members played a central role in healthcare interactions, sometimes facilitating but occasionally overshadowing patient voices. Organizational barriers, including high patient load and time constraints, limited SDM, while positive provider communication and continuity enabled engagement. Sociocultural factors included strong respect for medical authority and pluralistic health-seeking, with patients often reluctant to disclose alternative treatments to allopathic doctors. Exclusion from SDM was linked to dissatisfaction and poor adherence.

**Conclusion:**

Addressing these barriers and leveraging enablers will require coordinated efforts in communication, health literacy, family engagement, and culturally sensitive practice to advance person-centered care.

## 1 Introduction

Multimorbidity is defined as the coexistence of two or more chronic conditions in an individual (1, 2). It is a growing public health challenge worldwide and its prevalence is increasing (3–6). Globally, multimorbidity affects approximately 37% of adults, with prevalence rising to over half among individuals aged 60 years and older, and it is notably higher in women than in men (7).

As multimorbidity becomes more common, health systems are increasingly challenged to meet the needs of this population. As a consequence, health systems globally face the complex challenge of managing overlapping conditions, often requiring long-term, coordinated care (8). This problem is especially serious in countries like India, which is currently undergoing a significant demographic transition, with a rapidly expanding older adult population. Data from the Longitudinal Ageing Study in India (LASI) indicate that nearly half of individuals aged 60 years and above experience multimorbidity (9). Recent studies from Kerala report a multi-morbidity prevalence ranging from 39.8 to 45.4% among adults aged 30–69 years (10, 11) and 59.2% among older adults aged 60 years and above (12). The fast-growing older population and changing patterns of illness put extra pressure on health systems that already have limited resources (13, 14). This trend has profound implications for healthcare delivery, especially at the primary care level, where much of chronic disease management is initiated and maintained (15).

For older adults, these system pressures often translate into real-world difficulties. Older adults with multimorbidity often encounter fragmented healthcare services, complex treatment regimens, and the cumulative burden of illness, all of which can negatively impact their physical, emotional, and social well-being (16–18). The inherent complexity of multimorbidity challenges traditional, disease-specific, episodic, and clinician-driven models of care, necessitating a shift toward more integrated, holistic, and person-centered approaches (18–20).

In this context, shared decision-making (SDM) has emerged as a key strategy to advance person-centered care. SDM is a collaborative process in which healthcare professionals and patients work together to make healthcare decisions, integrating the best available clinical evidence with the patient’s values, preferences, and lived experiences (21). Many older individuals and their family members who provide care wish to actively participate in the decision-making process (22, 23). This approach respects patient autonomy and acknowledges the valuable insights that patients bring regarding their own health and priorities. Managing multimorbidity often requires making choices between different treatments and considering their effects on daily life (24, 25). By aligning healthcare decisions with patient values and preferences, SDM can enhance treatment adherence, reduce decisional conflict, and improve patient satisfaction (26, 27). Evidence also suggests that effective SDM is associated with improved health outcomes, greater patient empowerment, and more efficient use of healthcare resources (28, 29). Importantly, shared decision-making (SDM) helps advance the United Nations Sustainable Development Goals, especially SDG 3, which aims to ensure healthy lives and well-being for all at all ages (30). While SDM is not explicitly named in the SDGs, it is fundamental to achieving SDG 3 by promoting patient-centered care, improving health outcomes, and supporting universal health coverage (31, 32). By empowering older adults with multimorbidity to participate actively in their care, SDM enhances the quality and safety of care and fosters inclusive, participatory decision-making, key principles emphasized in the global SDG agenda (33). Thus, advancing SDM is not only central to person-centered care, but also to the broader pursuit of sustainable and equitable health systems.

However, despite its recognized benefits, SDM is not yet routinely practiced in many settings. Despite its inclusion in clinical guidelines and health policy frameworks in many high-income countries, the real-world implementation of SDM remains inconsistent, particularly in LMICs (34). In India, the adoption of SDM is limited, influenced by systemic constraints and sociocultural factors such as hierarchical doctor-patient relationships, time-pressured consultations, and a lack of institutional support for communication training and decision aids (35–39).

Kerala provides a unique context to examine these issues. In Kerala, the primary healthcare system serves as the first point of contact for a large segment of the population, particularly older adults in rural and semi-urban areas. Kerala is recognized for its robust public health infrastructure, high literacy rates, and progressive health indicators, and has one of the most rapidly aging populations in India (40, 41). Recent reforms, such as the transformation of primary health centers into family health centers, have aimed to strengthen primary care and promote patient-centered approaches (42, 43). Despite these advances, evidence suggests that community involvement in health decision-making remains limited, and older adults often defer to the authority of healthcare providers (44–46). Time constraints, provider workloads, and the absence of formal mechanisms for eliciting patient preferences further hinder the practice of SDM (47, 48).

To address these complex, multi-level barriers, a comprehensive framework is needed. Given the multifaceted nature of SDM, this study draws on the Socio-Ecological Model (SEM) as a guiding framework (49). The SEM recognizes that health behaviors and decisions are shaped by factors at multiple, interacting levels: individual, interpersonal, organizational, and broader sociocultural and policy contexts. Applying this framework allows for a comprehensive exploration of the barriers and facilitators to SDM among older adults with multimorbidity in Kerala’s primary care settings. Accordingly, this study aims to explore the experiences and perspectives of older adults with multimorbidity regarding SDM in Kerala’s primary care, identifying multi-level barriers and enablers.

## 2 Materials and methods

### 2.1 Study design and setting

This descriptive qualitative study design was employed. The study adhered to the Standards for Reporting Qualitative Research (SRQR) guidelines (50). Data was collected from four primary health centres (PHCs) in Kerala, namely Mynagapally, Punnapra, Kalloorkad, and Engandiyur. In the Indian health system, PHCs serve as the first point of contact for most patients, particularly in rural and semi-urban areas. They provide a comprehensive range of essential health services, including maternal and child health, immunizations, management of communicable and non-communicable diseases, health education, and referral services. In Kerala, PHCs play a pivotal role in chronic disease management through dedicated non-communicable disease (NCD) clinics, operated under the state’s “Amrutham Arogyam” program. This initiative focuses on early detection, screening, treatment, and the promotion of healthy lifestyles to address the rising burden of conditions such as diabetes and hypertension.

### 2.2 Participants

A purposive sampling strategy was employed to achieve maximum variation, ensuring representation across age, gender, socioeconomic background, and type of multimorbidity. This approach facilitated the inclusion of diverse perspectives and experiences related to shared decision-making in primary care.

Inclusion criteria were: (1) adults aged 60 years and above; (2) presence of multimorbidity, defined as the coexistence of two or more chronic non-communicable conditions (e.g., diabetes, cardiovascular disease, cancer, stroke, chronic obstructive pulmonary disease, or chronic kidney disease); and (3) ability to converse in either Malayalam or English. Exclusion criteria included: (1) diagnosis of dementia; and (2) communication impairments hindering meaningful participation.

Potential participants were identified by healthcare providers at the PHCs and approached by the research team after initial eligibility screening. Four interviews were conducted from each district, which sums up to a total of 16 interviews. Data collection proceeded iteratively until the lead researcher, in collaboration with academic supervisors, determined that data saturation was achieved (51). This decision was based on a systematic analysis of the interview data, regularly examining emerging themes, codes, and patterns. Saturation was reached when no new information or insights emerged, indicating further interviews would yield redundant data. The evaluation included monitoring recurring themes, verifying data redundancy, and confirming rich, comprehensive narratives from diverse participant perspectives.

### 2.3 Data collection

Individual in-depth semi-structured interviews were conducted with participants between June and December 2024. The interview guide, developed with reference to the SEM, included questions designed to elicit participants’ experiences and perspectives on SDM at the individual (e.g., health beliefs, literacy), interpersonal (e.g., family involvement), organizational (e.g., provider practices, continuity), and sociocultural/policy (e.g., cultural norms, health system factors) levels. The guide was pilot tested with five interviews and refined accordingly, the interviews were designed to last between 30 to 60 min, depending on the flow of conversation and the level of detail provided by the participants.

The interview questions followed a hierarchical structure, beginning with open-ended questions to allow participants to describe their general experiences. For example, an initial question asked: “What are the difficulties caused by multimorbidity in your life?” Subsequent follow-up questions were tailored to explore specific issues raised by participants, such as, “What obstacles do you face in managing the problems caused by your health conditions?” or “What kind of support would be most helpful from your family, community health centers, or other organizations in primary care settings?” To ensure thorough understanding and contextual validation, prompts were used when participants provided insufficiently detailed responses, such as, “How would the support you mentioned help you specifically?” This approach helped to validate the participants’ priorities and needs in their own words.

The interviews were held in person at the primary health centers (PHCs) or at the home of the participant, depending on the participant’s preference and convenience. Before each interview, the researcher thoroughly explained the purpose of the study and the interview process to the participants, ensuring they understood the content and objectives. The duration of each interview ranged from 45 to 60 min. During the interviews, the researcher remained respectful, objective, and flexible, adjusting the order of questions as needed and asking follow-up questions to probe deeper into specific responses. This approach ensured a more natural and fluid conversation, allowing participants to express their thoughts and experiences fully. The interviewer ensured privacy and that no other persons were present during interviews. Field notes were taken during and immediately after each interview to capture non-verbal cues and contextual information. The interviews were audio-recorded and transcribed verbatim. The Malayalam transcription then translated to English. Transcripts were not returned to participants, however, the researcher documented key points during the interviews and discussed these summaries with the participants to verify that they accurately reflected their experiences and perspectives. Participants were given the opportunity to provide feedback, clarify any misunderstandings, and offer additional insights. This process helped to enhance the credibility and authenticity of the study’s findings.

### 2.4 Reflexivity and positionality

The research team recognized the importance of reflexivity throughout the study. Data collection was conducted by the lead researcher, a male PhD scholar with a background in social work in public health and expertise in older adult care and management of non-communicable diseases. He has prior experience as a research associate on a qualitative study of health system preparedness for road traffic injuries. He has completed a four-credit course on social science research and qualitative methods. As a native of Kerala, the researcher’s familiarity with the local language, culture, and healthcare context facilitated rapport-building and contextual understanding during interviews with older adults. There was no prior relationship between the interviewer and participants.

To enhance the credibility and trustworthiness of the findings, the lead researcher maintained a reflexive journal to document personal assumptions, preconceptions, and observations throughout the research process. Regular supervisory meetings were held with three academic supervisors, who provided critical feedback and guidance on data collection, analysis, and interpretation. These discussions encouraged the researcher to critically examine how their background and positionality might influence interactions with participants and the interpretation of findings. This reflexive approach aimed to ensure that the analysis authentically represented participants’ perspectives, rather than being shaped by researcher expectations or prior experiences.

### 2.5 Data analysis

Thematic analysis was employed to analyze the interview data, following the six-step framework outlined by Braun and Clarke (52). These steps included: (1) familiarization with the data, (2) generating initial codes, (3) searching for themes, (4) reviewing themes, (5) defining and naming themes, and (6) producing the final report. Within 24 h of each interview, audio recordings were transcribed verbatim by the researcher. The interviews conducted in Malayalam were initially transcribed verbatim in the original language and subsequently translated into English by the lead researcher. To ensure the accuracy and quality of the translation, a second independent bilingual reviewer cross-checked the English transcripts against the original Malayalam audio recordings, resolving any discrepancies through consensus discussion. This process ensured that the nuances and meanings of participants’ expressions were faithfully preserved in the English translations.

The transcripts were then imported into NVivo 12 (53) software to facilitate systematic data management, coding, and retrieval. SEM was used as a sensitizing framework to organize and interpret themes across individual, interpersonal, organizational, and sociocultural/policy levels.

To ensure analytic rigor and consistency, the lead researcher conducted the initial coding of all transcripts. A subset of transcripts was independently reviewed by the three academic supervisors, who provided critical feedback and suggestions. Discrepancies or differences in coding were addressed through a process of contextual verification by revisiting the original transcripts, followed by collaborative discussions between the lead researcher and supervisors. Where necessary, consensus was reached through further deliberation. The codes were then refined and systematically organized into broader themes and sub-themes. The research team-comprising the lead researcher and supervisors jointly reviewed the relevance and coherence of the themes in relation to the study objectives and finalized their naming. This collaborative and reflexive process ensured a transparent and robust interpretation of the data, allowing for nuanced insights into the shared decision-making experiences of older adults with multimorbidity in primary care settings.

### 2.6 Ethics

This study was approved by the Institutional Ethics Committee (IEC) of Kasturba Medical College (KMC) and Kasturba Hospital (KH), Karnataka, India (Ethical Review No. IEC1: 05/2023, dated 27 July 2023). Administrative permission to conduct the study and collect data from primary health centers was obtained from the Directorate of Health Services, Kerala (Order No. MC4-48885/2022/DHS dated 09/2022). Written and verbal informed consent was obtained from all participants, ensuring that they understood their rights and voluntarily agreed to participate. All data collected was kept confidential and anonymous, and no identifying information was published in this paper.

## 3 Results

Researcher approached 19 possible participants where 16 of them agreed for the interview. The age of participants is in the range of 60 to 78 years, out of which eight are male and eight are female. 13 participants are staying with their family while the remaining are living alone. 10 participants are not working, three are unskilled labors, two are self-employed and a retired. 12 participants follow Allopathy while the remaining four rely both on Allopathy and Ayurveda (Tables 1, 2).

**Table 1:** Participant demographics.

ID	Age	Sex	Eduction	Income	District	Living Situation	Occupation	Main Health Conditions	Mode(s) of Treatment
P1	78	Male	Graduated	APL	Alappuzha	With family	Retired	Diabetes, High blood pressure, Cataract	Allopathy
P2	75	Female	Secondary	BPL	Alappuzha	With family	Self employed	Low blood pressure, Arthritis (back pain), Asthma	Allopathy and Ayurveda
P3	72	Female	Primary	APL	Alappuzha	With family	Not working	Arthritis, Low blood pressure, High cholesterol, Breathing issues	Allopathy and Ayurveda
P4	68	Female	Primary	APL	Alappuzha	With family	Not working	Diabetes, High blood pressure, High cholesterol, Vision issues	Allopathy
P5	60	Male	Primary	APL	Trissur	With family	Unskilled labor	Blood pressure, Diabetes	Allopathy and Ayurveda
P6	75	Male	Secondary	BPL	Trissur	With family	Not working	Diabetes, High blood pressure, High cholesterol, COPD (bypass surgery)	Allopathy
P7	67	Female	Secondary	APL	Trissur	With family	Not working	Diabetes, High blood pressure	Allopathy
P8	63	Female	Secondary	BPL	Trissur	Alone	Not working	Diabetes, High blood pressure, Arthritis, Kidney disease	Allopathy
P9	62	Female	Graduation	APL	Ernakulam	Alone	Not working	Diabetes, Kidney disease	Allopathy
P10	76	Male	Primary	BPL	Ernakulam	With family	Not working	Blood pressure, Stroke, Asthma	Allopathy
P11	60	Female	Secondary	APL	Ernakulam	With family	Not working	Cancer, Kidney disease	Allopathy
P12	65	Male	Primary	APL	Ernakulam	With family	Unskilled labor	Blood pressure, Asthma, Cataract, Hearing issues	Allopathy
P13	62	Male	Secondary	BPL	Kollam	With family	Unskilled labor	Diabetes, Blood pressure, Arthritis	Allopathy
P14	70	Male	Primary	BPL	Kollam	With family	Not working	Diabetes, Blood pressure, Arthritis, Neuro issues	Allopathy and Ayurveda
P15	69	Male	Secondary	APL	Kollam	With family	Not working	Diabetes, Blood pressure, Arthritis	Allopathy
P16	61	Female	Graduation	APL	Kollam	Alone	Self employed	Diabetes, Blood pressure, Arthritis, High cholesterol	Allopathy

**Table 2:** Themes and sub-themes.

SEM Level	Theme	Sub-Themes
Individual Level	Health Literacy and Self-Efficacy	- Difficulty understanding medical terms - Hesitancy to ask questions - Passive participation
Interpersonal Level	Family Involvement and Influence	- Family as support in healthcare interactions - Family sometimes overshadowing patient preferences - Not strongly gendered in Kerala
	Respect for Medical Authority	- Deference to doctors - Reluctance to question or challenge recommendations
Organizational Level	Provider Communication and Continuity	- Value of clear explanations and empathy - Importance of continuity of care - Trust and partnership
	Time Constraints and System Pressures	- High patient load - Rushed consultations - Limited opportunity for dialogue
Sociocultural/ Policy-Level	Community Norms and Pluralistic Health-Seeking	- Respect for medical authority - Use of both allopathic and alternative medicine - Reluctance to disclose alternative treatment to allopathic doctors
Cross-Cutting	Consequences of Exclusion	- Dissatisfaction with care - Confusion about treatment plans - Poor adherence to treatment

### 3.1 Individual-level factors

#### 3.1.1 Health literacy and self-efficacy

A substantial number of participants described significant challenges in understanding their medical conditions, treatment options, and the implications of various choices. Many expressed uncertainties about medical terminology and a lack of confidence in their ability to actively participate in healthcare discussions. For some, the language used by providers was perceived as too technical or unfamiliar, which led them to withdraw from conversations and defer to the provider’s expertise.

Participant 13: *“I often feel lost when the doctor talks about my illnesses. I do not understand many things they say, so I just listen quietly. I ask about the medicine and when to have it and how many times a day.”*

Another participant 3: *“They write prescriptions, sometimes nobody can read. Then I do not understand which tablet is for what purpose. Pharmacists also do not have time to explain. I cannot read it, so I have to ask my neighbour.”*

This lack of understanding often resulted in a passive approach to healthcare, where participants simply accepted whatever was recommended without seeking clarification or additional information. Participants reported feeling hesitant to ask questions or clarify doubts, fearing that they might appear ignorant or disrespectful to the provider.

Participant 8: *“I worry the doctor will think I am wasting his time if I ask too many questions. What if I am asking the wrong questions? Now a days, there is no talk, it is just looking at the test results and getting signed in my notebook (prescription book).*

Participant 5: *“What will the other patients who are waiting in the queue think? Sometimes if we spend more time with the doctor, people start yelling. So, I keep my doubts to myself.”*

Such experiences contributed to a sense of helplessness and resignation, with many describing themselves as “just following instructions” rather than being active partners in their care. This limited health literacy and self-efficacy were seen as major barriers to meaningful involvement in SDM.

### 3.2 Interpersonal-level factors

#### 3.2.1 Family involvement and influence

Family members, particularly adult children and spouses played a prominent role in mediating interactions with healthcare providers. Many older adults were accompanied by relatives to appointments, with family members often taking the lead in discussions and, at times, making decisions on behalf of the patient. This dynamic was especially evident among participants who felt less confident in their own ability to communicate with healthcare professionals or who had limited health literacy.

Participant 11: *“Usually, my son comes with me to the clinic. He talks to the doctor and explains things to me later. He knows better than me.”*

Participant 7: *“My husband always comes with me to the health center. He listens to what the doctor says. He reminds me about the medicines and when to go for check-ups.”*

While this support was appreciated, especially by those with physical or cognitive limitations, some participants felt their own preferences were overlooked or overshadowed by family opinions. The presence of family could be both empowering and constraining, depending on the nature of relationships and the degree of respect for the older adult’s autonomy.

Participant 2: *“My daughter is always there to support me. I tell her what I feel. She answers the questions of doctor. It’s easier to let her handle everything.”*

While family members, including adult children and spouses, often played a central role in mediating interactions with healthcare providers, this dynamic was not strongly linked to gender in our setting. Both men and women described relying on family for support and sometimes deferring to relatives in discussions with providers.

#### 3.2.2 Respect for medical authority

A deeply ingrained respect for doctors and medical authority was evident across many interviews. Participants described doctors as the ultimate decision-makers and expressed reluctance to question or challenge their recommendations. For many, the physician’s expertise was seen as unquestionable, and their own role was to comply rather than collaborate.

Participant 6: *“We have always believed that the doctor knows best. Who am I to question what he says?”*

Some participants felt that voicing concerns or preferences might be seen as disrespectful or ungrateful, leading them to remain silent even when they had doubts or preferences. This difference to medical authority contributed to a largely passive role for patients in the decision-making process, reinforcing a traditional, paternalistic model of care.

Participant 3: *“I do not want to offend the doctor by disagreeing. Sometimes my ayurveda doctor suggest me to do things differently and I get confused. But I do not ask the doctor. He is the expert, after all. What if he does not like.”*

Such attitudes were often shaped by lifelong experiences and reinforced by community norms, making it challenging for patients to assert their preferences even when they wished to do so.

### 3.3 Organizational-level factors

#### 3.3.1 Provider communication and continuity

Positive experiences of SDM were closely linked to providers who took the time to explain treatment options, encouraged questions, and demonstrated empathy. Participants valued healthcare professionals who remembered their history and engaged them in meaningful conversations about their care. These interactions fostered a sense of trust, comfort, and partnership, making it easier for patients to express their concerns and preferences.

Participant 14: *“The doctor who knows me well always asks how I’m feeling and explains the different options. It makes me feel like my opinion matters.”*

Continuity of care-consistently seeing the same provider over time-was identified as a key facilitator of trust and more collaborative interactions. Participants who experienced continuity reported greater satisfaction and a stronger sense of agency in their care.

Participant 9: *“When I see the same doctor every time, I feel more comfortable talking about my problems. He understands my situation better.”*

However, such positive experiences were not universal. Many participants described brief, transactional encounters in which providers focused on issuing prescriptions rather than engaging in dialogue. The lack of continuity and personalized attention often left patients feeling like just another number in a busy clinic.

Participant 1: *“Most of the time, the doctor just writes the prescription and moves on to the next patient. There’s no time for discussion.”*

#### 3.3.2 Time constraints and system pressures

High patient volumes and limited consultation times were frequently cited as barriers to SDM. Participants described rushed appointments, with little opportunity to ask questions or discuss preferences. The pressure on providers to see many patients in a short period often resulted in consultations that were hurried and impersonal.

Participant 6: *“The waiting room is always full, and the doctor is in a hurry. I feel bad taking up more of his time, so I just listen to what he says. Sometimes I do not fully understand. Then I ask the pharmacist.”*

Some participants perceived that providers were under pressure to see as many patients as possible, which further limited opportunities for meaningful engagement. This organizational reality reinforced a sense of being “processed” rather than cared for, and discouraged patients from seeking clarification or voicing their concerns.

Participant 16: *“I can see the doctor is busy. I do not want to trouble him with my worries. When doctor spend more time with a patient, other people will start making noise. Everyone wants to get it done as soon as possible.”*

These organizational constraints contributed to a sense of frustration and resignation among patients, who felt that the system did not allow for genuine dialogue or partnership.

### 3.4 Community norms and pluralistic health-seeking

Several participants described feeling uncertain or reluctant to disclose to their allopathic doctor that they were also receiving Ayurvedic or other alternative treatments. This hesitancy often stemmed from concerns about being judged, misunderstood, or admonished by the allopathic provider.

Participant 14: *“I take ayurvedic medicine for my joint pain, but I do not tell my allopathy doctor because I’m afraid he will ask me to stop or say something negative.”*

In contrast, participants felt more comfortable telling their Ayurveda practitioner that they were also seeing an allopathic doctor, reflecting a perception that Ayurveda is more accepting of integrative approaches.

Participant 5: *“When I go to the Ayurveda doctor, I can say I am taking tablets from the health centre. They usually ask about it and do not mind.”*

This selective disclosure highlights a subtle but important aspect of patient-provider relationships in Kerala: while patients value the expertise of allopathic doctors, they may withhold information about alternative treatments to avoid conflict or disapproval. Such practices can have implications for safety, drug interactions, and the effectiveness of care. At the same time, the coexistence of multiple medical systems in Kerala fosters a unique environment where patients navigate between traditions, sometimes blending approaches to suit their needs.

### 3.5 Consequences of exclusion

Participants who felt excluded from decision-making reported a range of negative outcomes, including dissatisfaction with care, confusion about their treatment plans, and poor adherence to prescribed regimens. When patients did not understand the rationale for their treatments or felt their concerns were not addressed, they were less likely to follow through with recommendations.

Participant 7: *“Sometimes I do not take the tablets because I do not really understand why I need them. No one explained it to me.”*

Others described feeling anxious, isolated, or disempowered, which affected their willingness to seek care or follow through with recommendations. The lack of explanation or involvement in decisions often led to uncertainty and disengagement, potentially undermining the effectiveness of care.

Participant 10: *“When nobody explains things to me, I feel lost and worried. It makes me afraid to go to the clinic.”*

### 3.6 Summary

The experiences of older adults with multimorbidity in Kerala’s primary care settings reveal a complex interplay of individual knowledge, family involvement, provider practices, and cultural norms shaping shared decision-making. While some participants described positive, inclusive encounters, many faced significant barriers at multiple SEM levels. These findings underscore the need for multi-level interventions to promote person-centered care and empower older adults to participate actively in decisions about their health.

## 4 Discussion

The SEM highlights how SDM is shaped by a dynamic interplay of individual, interpersonal, organizational, and sociocultural/policy factors. Our findings reveal that while there are positive examples of participatory care, substantial barriers persist at every level, echoing patterns observed across low- and middle-income countries (LMICs) (54). Shared decision-making has important implications for the attainment of the Sustainable Development Goals (SDGs). Achieving this goal requires not only access to services, but also meaningful patient engagement and participation in care decisions as it promotes patient empowerment, improves treatment adherence, and enhances health outcomes.

### 4.1 Individual-level factors

At the individual level, limited health literacy and low self-efficacy emerged as critical barriers to active engagement in SDM. Many participants described difficulties in understanding medical terminology, treatment options, and the implications of various choices. This uncertainty often resulted in a passive approach, with patients deferring decisions to healthcare providers rather than actively participating in their own care. These findings align with previous research in LMICs, which has consistently highlighted low health literacy as a key obstacle to SDM (28, 54–56). Additionally, some participants expressed apprehension about asking questions, fearing they might be seen as challenging the provider’s authority or wasting their time. This lack of confidence and perceived power imbalance further limited their willingness to engage in meaningful dialogue. To address these barriers, targeted health education interventions and the use of patient-friendly decision aids are essential. Providing information in clear, accessible language and encouraging patients to voice their concerns can empower older adults to take a more active role in their healthcare decisions (57). Decision aids and pictorial tools have been shown to effectively reduce decisional conflict and support shared decision-making in LMICs, with evidence from Malaysia highlighting the importance of addressing cultural paternalism and role boundaries among healthcare providers for successful implementation. In India, a self-administered, adaptive decision aid significantly lowered decisional conflict among early breast cancer patients, demonstrating feasibility and cultural adaptability in improving patient engagement and preference-concordant surgical decisions (58, 59).

### 4.2 Interpersonal-level factors

Family involvement was a defining feature of SDM in Kerala. Family members, especially adult children, frequently accompanied older adults to appointments, mediated interactions with providers, and sometimes made decisions on the patient’s behalf. While this support was often valued, particularly by those with physical or cognitive limitations, it could also overshadow the patient’s own preferences. Notably, in contrast to findings from other Indian states and some LMICs, this dynamic in Kerala was not strongly gendered, reflecting the state’s relatively high levels of gender equity and autonomy among older women.

Our findings that family members can both enable, and hinder decision-making are consistent with broader research on family-centered care (60, 61). On one hand, family members can help patients articulate concerns, interpret complex information, and provide emotional support. On the other, their involvement may inadvertently suppress the patient’s voice, especially if family members dominate discussions or make decisions without fully consulting the older adult. Effective SDM in such contexts requires a nuanced approach that respects patient autonomy while constructively integrating family input. Training providers to facilitate inclusive conversations and explicitly inviting the patient’s perspective can help balance these influences.

### 4.3 Organizational-level factors

At the organizational level, provider communication and continuity of care emerged as critical enablers of SDM. Participants who consistently saw the same provider and experienced empathetic, clear communication described feeling more included in decisions about their care. These positive encounters fostered trust, comfort, and a sense of partnership, making it easier for patients to express their concerns and preferences. However, high patient loads, limited consultation times, and system pressures often resulted in rushed, transactional encounters that limited opportunities for meaningful engagement. Many participants described feeling like “just another number” in a busy health center, with little time or space to discuss their preferences or ask questions. These challenges are widely reported in LMIC settings, where resource constraints and workforce shortages are common (62, 63). Such organizational realities reinforce passive patient roles and undermine the principles of SDM. Interventions at this level should focus on providing training in SDM techniques, restructuring clinic workflows to allow more time for patient engagement, and promoting continuity of care. Studies have shown that even brief SDM interventions, when consistently applied, can improve patient satisfaction and health outcomes (64, 65).

### 4.4 Sociocultural and policy-level factors

Kerala’s community norms strongly emphasize respect for medical authority, which can discourage patients from questioning providers or expressing preferences. This cultural expectation of difference to expertise was evident in many participants’ accounts, regardless of gender or educational background. While such respect can foster trust, it may also limit open dialogue and reduce opportunities for SDM (66, 67).

A unique finding in this context was the prevalence of pluralistic health-seeking, with many patients consulting both allopathic and Ayurvedic practitioners. Several participants hesitated to disclose their use of ayurveda to allopathic doctors, fearing disapproval or negative reactions. This selective disclosure may have implications for treatment safety, drug interactions, and the effectiveness of SDM, highlighting the need for open, nonjudgmental communication and provider awareness of pluralistic practices (68, 69). At the policy level, Kerala’s health reforms have strengthened primary care and promoted patient-centered approaches, yet the real-world implementation of SDM remains inconsistent. Ongoing system-level support and investment are needed to institutionalize SDM practices and create an environment conducive to participatory care (70).

### 4.5 Consequences of exclusion

Exclusion from SDM was associated with dissatisfaction, confusion about treatment plans, and poor adherence to prescribed regimens. When patients did not understand the rationale for their treatments or felt their concerns were not addressed, they were less likely to follow through with recommendations. This finding is supported by a substantial body of evidence that SDM improves adherence, self-management, and health outcomes in chronic disease management (28). For older adults with multimorbidity, SDM is not only an ethical imperative but also a practical strategy to improve outcomes, as patients’ willingness to adhere to treatment is closely linked to their understanding and involvement in care decisions.

### 4.6 Implications for policy and practice

Individual level and Interpersonal level: Implement educational programs and initiatives to promote and encourage the adoption of shared decision-making among both patients and primary caregivers.

Organizational level: Training initiatives for primary healthcare providers should emphasize communication and skills to encourage SDM tailored to the specific context of Kerala’s healthcare system. Additionally, clinic workflows should be redesigned to extend consultation durations, helping to alleviate the effects of high patient volumes and brief appointments. Enhancing continuity of care can also build patient trust and improve adherence to treatment plans.

Sociocultural and policy levels: State health policies should formally integrate SDM practices into primary care within the framework of Kerala’s ongoing healthcare reforms. Encouraging open communication about diverse health-seeking behaviors, including both allopathic and traditional medicine, can help address patient concerns and enhance transparency in treatment. Additionally, policies should ensure creation of a space for patients to openly share their use of alternative medicine.

## 5 Conclusion

Interpreting the findings through the Socio-Ecological Model reveals that SDM for older adults with multimorbidity in Kerala is influenced by a complex interplay of individual, interpersonal, organizational, and sociocultural factors. Addressing these multi-level barriers and leveraging enablers will require coordinated efforts at all levels of the health system. Interventions that prioritize communication, education, family engagement, and culturally sensitive practice are essential for advancing person-centered care and improving health outcomes in this vulnerable population.

## Data Availability

The raw data supporting the conclusions of this article will be made available by the authors, without undue reservation.

## References

[1] Almirall J Fortin M. The coexistence of terms to describe the presence of multiple concurrent diseases. J Comorbidity. (2013) 3:4–9. doi: 10.15256/joc.2013.3.22 PMC5636023 29090140

[2] Van Den Akker M Buntinx F Knottnerus JA. Comorbidity or multimorbidity: what’s in a name? A review of literature. Eur J Gen Pract. (1996) 2:65–70. doi: 10.3109/13814789609162146

[3] Arokiasamy P Uttamacharya U Jain K. Multi-morbidity, functional limitations, and self-rated health among older adults in India: cross-sectional analysis of LASI pilot survey, 2010. SAGE Open. (2015) 5:640. doi: 10.1177/2158244015571640

[4] McKeown RE. The epidemiologic transition: changing patterns of mortality and population dynamics. Am J Lifestyle Med. (2009) 3:19S–26S. doi: 10.1177/1559827609335350, PMID: 20161566 PMC2805833

[5] Uijen A van de Lisdonk E. Multimorbidity in primary care: prevalence and trend over the last 20 years. Eur J Gen Pract. (2008) 14:28–32. doi: 10.1080/13814780802436093 18949641

[6] Violan C Foguet-Boreu Q Flores-Mateo G Salisbury C Blom J Freitag M. Prevalence, determinants and patterns of multimorbidity in primary care: A systematic review of observational studies. PLoS One. (2014) 9:149. doi: 10.1371/journal.pone.0102149, PMID: 25048354 PMC4105594

[7] Chowdhury SR Chandra Das D Sunna TC Beyene J Hossain A. Global and regional prevalence of multimorbidity in the adult population in community settings: a systematic review and meta-analysis. EClinicalMedicine. (2023) 57:101860. doi: 10.1016/j.eclinm.2023.101860 36864977 PMC9971315

[8] Moffat K Mercer SW. Challenges of managing people with multimorbidity in today’s healthcare systems. BMC Fam Pract. (2015) 16:129. doi: 10.1186/s12875-015-0344-4 26462820 PMC4604728

[9] International Institute for Population Sciences, NPHCE, HSPH and USC. Longitudinal Ageing Study in India (LASI) Wave-1. India: National Programme for Health Care of Elderly (NPHCE), MoHFW, Harvard T. H. Chan School of Public Health (HSPH) and the University of Southern California (USC) (2020).

[10] Ismail S Stanley A Jeemon P. Prevalence of multimorbidity and associated treatment burden in primary care settings in Kerala: a cross-sectional study in Malappuram District, Kerala, India. Wellcome Open Res. (2022) 7:67. doi: 10.12688/wellcomeopenres.17674.2, PMID: 35592547 PMC9086527

[11] Jeemon P Rohini C. Prevalence and patterns of multi-morbidity in the productive age group of 30-69 years: a cross-sectional study in Pathanamthitta District, Kerala. Wellcome Open Res. (2020) 5:233. doi: 10.12688/wellcomeopenres.16326.2 33215050 PMC7658726

[12] Patel P Muhammad T Sahoo H. The burden of disease-specific multimorbidity among older adults in India and its states: evidence from LASI. BMC Geriatr. (2023) 23:53. doi: 10.1186/s12877-023-03728-1, PMID: 36710322 PMC9885687

[13] Basto-Abreu A Barrientos-Gutierrez T Wade AN Oliveira de Melo D Semeão de Souza AS Nunes BP. Multimorbidity matters in low and middle-income countries. J Multimorb Comorb. (2022) 12:26335565221106074. doi: 10.1177/26335565221106074 35734547 PMC9208045

[14] Varanasi R Sinha A Bhatia M Nayak D Manchanda RK Janardhanan R. Epidemiology and impact of chronic disease multimorbidity in India: a systematic review and meta-analysis. J Multimorb Comorb. (2024) 14:26335565241258851. doi: 10.1177/26335565241258851 38846927 PMC11155324

[15] Skou ST Mair FS Fortin M Guthrie B Nunes BP Miranda JJ. Multimorbidity. Nat Rev Dis Primers. (2022) 8:48. doi: 10.1038/s41572-022-00376-4, PMID: 35835758 PMC7613517

[16] Akintayo-Usman NO. Fragmentation of care: a major challenge for older people living with multimorbidity. Geriatr Gerontol Aging. (2021) 15:e0210030. doi: 10.53886/gga.0210030

[17] Lee JE Lee J Shin R Oh O Lee KS. Treatment burden in multimorbidity: an integrative review. BMC Prim Care. (2024) 25:352. doi: 10.1186/s12875-024-02586-z, PMID: 39342121 PMC11438421

[18] Zhao L Chang B Hu Q Chen X Du J Shao S. The health care needs of multidimensional frail elderly patients with multimorbidity in primary health-care settings: a qualitative study. BMC Primary Care. (2025) 26:128. doi: 10.1186/s12875-025-02836-8, PMID: 40281402 PMC12032638

[19] van Blarikom E Fudge N Swinglehurst D. The emergence of multimorbidity as a matter of concern: a critical review. BioSocieties. (2023) 18:614–31. doi: 10.1057/s41292-022-00285-5

[20] Bogerd MJ Slottje P Bont J Van Hout HP. Development of a person-centred care approach for persons with chronic multimorbidity in general practice by means of participatory action research. BMC Prim Care. (2024) 25:114. doi: 10.1186/s12875-024-02364-x, PMID: 38627610 PMC11020638

[21] Chen M Sepucha K Bozic KJ Jayakumar P. Value-based healthcare: integrating shared decision-making into clinical practice. Clin Orthop Relat Res. (2023) 481:448–50. doi: 10.1097/CORR.0000000000002580, PMID: 36735904 PMC9928684

[22] Chewning B Bylund CL Shah B Arora NK Gueguen JA Makoul G. Patient preferences for shared decisions: A systematic review. Patient Educ Couns. (2012) 86:9–18. doi: 10.1016/j.pec.2011.02.004, PMID: 21474265 PMC4530615

[23] Wolff JL Boyd CM. A look at person-centered and family-centered care among older adults: results from a national survey. J Gen Intern Med. (2015) 30:1497–504. doi: 10.1007/s11606-015-3359-6, PMID: 25933625 PMC4579212

[24] Hoffmann T Jansen J Glasziou P. The importance and challenges of shared decision making in older people with multimorbidity. PLoS Med. (2018) 15:e1002530. doi: 10.1371/journal.pmed.1002530, PMID: 29534067 PMC5849298

[25] Shi S Liu X Li Y Yang C. Shared decision making for people living with multimorbidity: A concept analysis. Patient Educ Couns. (2025) 135:108712. doi: 10.1016/j.pec.2025.108712, PMID: 40120465

[26] Fiorillo A Barlati S Bellomo A Corrivetti G Nicolò G Sampogna G. The role of shared decision-making in improving adherence to pharmacological treatments in patients with schizophrenia: a clinical review. Ann General Psychiatry. (2020) 19:43. doi: 10.1186/s12991-020-00293-4, PMID: 32774442 PMC7409631

[27] Hoque F. Shared decision-making in patient care: advantages, barriers and potential solutions. J Brown Hosp Med. (2024) 3:13–5. doi: 10.56305/001c.122787, PMID: 40028803 PMC11864379

[28] Klein LW. Shared decision-making: the more the patient knows, the better the decision that is made. Rev Cardiovasc Med. (2023) 24:8232. doi: 10.31083/j.rcm2408232, PMID: 39076709 PMC11262446

[29] Okunrintemi V Valero-Elizondo J Stone NJ Blankstein R Blaha MJ Gulati M. Shared decision making and patient reported outcomes among adults with atherosclerotic cardiovascular disease, medical expenditure panel survey 2006–2015. Am J Prev Cardiol. (2021) 8:100281. doi: 10.1016/j.ajpc.2021.100281, PMID: 34877558 PMC8627957

[30] United Nations. Ensure healthy lives and promote well-being for all at all ages. New York: United Nations (2016).

[31] Komatsu Y. Polypharmacy and sustainable developmental goals: linking evidence-based medicine, patient engagement, and shared decision-making. Ren Replace Ther. (2023) 9:20. doi: 10.1186/s41100-023-00474-3

[32] Nunes AR Lee K O’Riordan T. The importance of an integrating framework for achieving the sustainable development goals: the example of health and well-being. BMJ Glob Health. (2016) 1:e000068. doi: 10.1136/bmjgh-2016-000068 PMC5321355 28588955

[33] Gera R Narwal R Jain M Taneja G Gupta S. Sustainable development goals: leveraging the global agenda for driving health policy reforms and achieving universal health coverage in India. Indian J Community Med. (2018) 43:255–9. doi: 10.4103/ijcm.IJCM_41_18, PMID: 30662175 PMC6319280

[34] Shaker MS Verdi M. Operationalizing shared decision making in clinical practice. Allergy Asthma Proc. (2024) 45:398–403. doi: 10.2500/aap.2024.45.240048, PMID: 39517076

[35] Doval D Kumar P Talwar V Vaid A Desai C Ostwal V. Shared decision-making and medicolegal aspects: delivering high-quality cancer care in India. Indian J Palliat Care. (2020) 26:405. doi: 10.4103/IJPC.IJPC_237_19, PMID: 33623298 PMC7888410

[36] Gravel K Légaré F Graham ID. Barriers and facilitators to implementing shared decision-making in clinical practice: a systematic review of health professionals’ perceptions. Implement Sci. (2006) 1:16. doi: 10.1186/1748-5908-1-16, PMID: 16899124 PMC1586024

[37] Pel-Littel RE Snaterse M Teppich NM Buurman BM van Etten-Jamaludin FS van Weert JCM. Barriers and facilitators for shared decision making in older patients with multiple chronic conditions: a systematic review. BMC Geriatr. (2021) 21:112. doi: 10.1186/s12877-021-02050-y, PMID: 33549059 PMC7866443

[38] Waddell A Lennox A Spassova G Bragge P. Barriers and facilitators to shared decision-making in hospitals from policy to practice: a systematic review. Implement Sci. (2021) 16:74. doi: 10.1186/s13012-021-01142-y, PMID: 34332601 PMC8325317

[39] Joseph L Greenfield S Manaseki-Holland S L TR S S Panniyammakal J. Patients’, carers’ and healthcare providers’ views of patient-held health records in Kerala, India: a qualitative exploratory study. Health Expect. (2023) 26:1081–95. doi: 10.1111/hex.13721 36782391 PMC10154823

[40] Rajan SI Shajan A Sunitha S. Ageing and elderly care in Kerala. China Rep. (2020) 56:354–73. doi: 10.1177/0009445520930393

[41] State Planning Board. Economic review 2017. Thiruvananthapuram, Kerala: State Planning Board (2017).

[42] Elamon J Franke RW Ekbal B. Decentralization of health services: the Kerala people’s campaign. Int J Health Serv. (2004) 34:681–708. doi: 10.2190/4L9M-8K7N-G6AC-WEHN, PMID: 15560430

[43] Krishnan A Varma RP Kamala R Anju R Vijayakumar K Sadanandan R. Re-engineering primary healthcare in Kerala. Public Health Action. (2023) 13:19–25. doi: 10.5588/pha.22.0033, PMID: 36949746 PMC9983803

[44] Sreekumar S Mishra S. Between aspirations and realities of participation: understanding the meanings of community participation in the context of family health Centre policy of Kerala. SSM Health Sys. (2024) 3:100023. doi: 10.1016/j.ssmhs.2024.100023

[45] Chi WC Wolff J Greer R Dy S. Multimorbidity and decision-making preferences among older adults. Ann Fam Med. (2017) 15:546–51. doi: 10.1370/afm.2106, PMID: 29133494 PMC5683867

[46] Sherwin S Winsby M. A relational perspective on autonomy for older adults residing in nursing homes. Health Expect. (2011) 14:182–90. doi: 10.1111/j.1369-7625.2010.00638.x, PMID: 21029285 PMC5060573

[47] Caverly TJ Hayward RA. Dealing with the lack of time for detailed shared decision-making in primary care: everyday shared decision-making. J Gen Intern Med. (2020) 35:3045–9. doi: 10.1007/s11606-020-06043-2, PMID: 32779137 PMC7572954

[48] Fusiak J Sarpari K Ma I Mansmann U Hoffmann VS. Practical applications of methods to incorporate patient preferences into medical decision models: a scoping review. BMC Med Inform Decis Mak. (2025) 25:109. doi: 10.1186/s12911-025-02945-5, PMID: 40033306 PMC11877743

[49] Bronfenbrenner U. The ecology of human development: experiments by nature and design. Cambridge, MA: Harvard University Press (1979).

[50] O’Brien BC Harris IB Beckman TJ Reed DA Cook DA. Standards for reporting qualitative research. Acad Med. (2014) 89:1245–51. doi: 10.1097/acm.0000000000000388, PMID: 24979285

[51] Guest G Bunce A Johnson L. How many interviews are enough? Field Methods. (2006) 18:59–82. doi: 10.1177/1525822x05279903

[52] Braun V Clarke V. Using thematic analysis in psychology. Qual Res Psychol. (2006) 3:77–101. doi: 10.1191/1478088706qp063oa

[53] NVivo. QSR IPL NVivo (Version 12). London: Sage Publications Ltd (2018).

[54] Sam S Sharma R Corp N Igwesi-Chidobe C Babatunde OO. Shared decision making in musculoskeletal pain consultations in low- and middle-income countries: a systematic review. Int Health. (2020) 12:455–71. doi: 10.1093/inthealth/ihz077, PMID: 31728513 PMC11701107

[55] Suppiah SD Malhotra R Tan YW Jessup RL Chew LST Tang WE. Prevalence of health literacy and its correlates from a national survey of older adults. Res Social Adm Pharm. (2023) 19:906–12. doi: 10.1016/j.sapharm.2023.02.013, PMID: 36898905

[56] Yogesh M Makwana N Trivedi N Damor N. Multimorbidity, health literacy, and quality of life among older adults in an urban slum in India: a community-based cross-sectional study. BMC Public Health. (2024) 24:1833. doi: 10.1186/s12889-024-19343-7, PMID: 38982428 PMC11234527

[57] Vogel A Guinemer C Fürstenau D. Patients’ and healthcare professionals’ perceived facilitators and barriers for shared decision-making for frail and elderly patients in perioperative care: a scoping review. BMC Health Serv Res. (2023) 23:197. doi: 10.1186/s12913-023-09120-4, PMID: 36829131 PMC9960423

[58] Joshi S Ramarajan L Ramarajan N Lee SS Deshpande O Fernandes E. Effectiveness of a decision aid plus standard Care in Surgical Management among Patients with Early Breast Cancer: A randomized clinical trial. JAMA Netw Open. (2023) 6:e2335941. doi: 10.1001/jamanetworkopen.2023.35941, PMID: 37782500 PMC10546236

[59] Tong WT Lee YK Ng CJ Lee PY. Factors influencing implementation of a patient decision aid in a developing country: an exploratory study. Implement Sci. (2017) 12:40. doi: 10.1186/s13012-017-0569-9, PMID: 28327157 PMC5361724

[60] Dijkman BL Luttik ML Van der Wal-Huisman H Paans W van Leeuwen BL. Factors influencing family involvement in treatment decision-making for older patients with cancer: A scoping review. J Geriatr Oncol. (2022) 13:391–7. doi: 10.1016/j.jgo.2021.11.003, PMID: 34776380

[61] Yang Y Qu T Yang J Ma B Leng A. Confucian Familism and shared decision making in end-of-life Care for Patients with advanced cancers. Int J Environ Res Public Health. (2022) 19:10071. doi: 10.3390/ijerph191610071, PMID: 36011706 PMC9408283

[62] Phelan H Yates V Lillie E. Challenges in healthcare delivery in low- and middle-income countries. Anaesth Intensive Care Med. (2022) 23:501–4. doi: 10.1016/j.mpaic.2022.05.004

[63] Mehta V Ajmera P Kalra S Miraj M Gallani R Shaik RA. Human resource shortage in India’s health sector: a scoping review of the current landscape. BMC Public Health. (2024) 24:1368. doi: 10.1186/s12889-024-18850-x, PMID: 38773422 PMC11110446

[64] Veenendaal HV Chernova G Bouman CM Etten-Jamaludin FSV Dieren SV Ubbink DT. Shared decision-making and the duration of medical consultations: a systematic review and meta-analysis. Patient Educ Couns. (2023) 107:107561. doi: 10.1016/j.pec.2022.11.003 36434862

[65] Lee PY Ng CJ. Practising shared decision making in primary care. Malays Fam Physician. (2021) 16:2–7. doi: 10.51866/cm0001, PMID: 33948136 PMC8088742

[66] Nimmon L Stenfors-Hayes T. The “handling” of power in the physician-patient encounter: perceptions from experienced physicians. BMC Med Educ. (2016) 16:114. doi: 10.1186/s12909-016-0634-0, PMID: 27091146 PMC4835893

[67] Charles C Gafni A Whelan T O’Brien MA. Cultural influences on the physician–patient encounter: the case of shared treatment decision-making. Patient Educ Couns. (2006) 63:262–7. doi: 10.1016/j.pec.2006.06.018, PMID: 17000073

[68] Rudra S Kalra A Kumar A Joe W. Utilization of alternative systems of medicine as health care services in India: evidence on AYUSH care from NSS 2014. PLoS One. (2017) 12:e0176916. doi: 10.1371/journal.pone.0176916, PMID: 28472197 PMC5417584

[69] Foley H Steel A Cramer H Wardle J Adams J. Disclosure of complementary medicine use to medical providers: a systematic review and meta-analysis. Sci Rep. (2019) 9:1573. doi: 10.1038/s41598-018-38279-8, PMID: 30733573 PMC6367405

[70] Prajitha KC Rahul A Chintha S Soumya G Maheswari Suresh M Nalina Kumari ANK. Strategies and challenges in Kerala’s response to the initial phase of COVID-19 pandemic: a qualitative descriptive study. BMJ Open. (2021) 11:e051410. doi: 10.1136/bmjopen-2021-051410 PMC8275365 34244285

